# Does young age influence the prognosis of colorectal cancer: a population-based analysis

**DOI:** 10.1186/1477-7819-12-370

**Published:** 2014-12-02

**Authors:** Andrew McKay, Jeniva Donaleshen, Ramzi M Helewa, Jason Park, Debrah Wirtzfeld, David Hochman, Harminder Singh, Donna Turner

**Affiliations:** Department of Surgery, University of Manitoba, GF-441, 820 Sherbrook St, Winnipeg, MB R3A 1R9 Canada; CancerCare Manitoba, 675 McDermot Avenue, Winnipeg, MB R3E 0V9 Canada; Faculty of Medicine, University of Manitoba, 260 Brodie Centre - 727 McDermot Avenue, Winnipeg, Manitoba R3E 3P5 Canada; Department of Medicine, University of Manitoba, Health Sciences Centre, Room GC425, 820 Sherbrook Street, Winnipeg, MB R3T 2N2 Canada

## Abstract

**Background:**

Controversy exists whether young patients diagnosed with colorectal cancer have a poorer prognosis. Although younger patients are more likely to have certain poor prognostic factors, prior studies have shown mixed results in terms of overall prognosis, which may be due to lack of adjustment for confounding factors. The primary objective of our study was to determine the effect of age on survival following treatment of colorectal cancer in the Province of Manitoba, Canada, while controlling for important cofactors.

**Methods:**

This was a population-based analysis of all adult patients (age ≥18 years) diagnosed with adenocarcinoma of the colon or rectum between 1 January 2004 and 31 December 2006 in the Province of Manitoba. Patient, tumor, and treatment factors were identified using administrative data. Five-year Kaplan-Meier survival and Cox proportional hazards model were analyzed to determine whether young age (45 years of age or younger) was associated with a poorer prognosis, while controlling for confounding variables.

**Results:**

Of the 2,086 patients identified, 70 (3.36%) were considered young. These patients were more likely to have T4 tumors and node-positive disease. Older patients had more advanced comorbidities. Young age was an independent predictor of better survival. Poorer survival was associated with male gender, increasing stage, higher grade, comorbidity, lower socioeconomic status, and lack of receipt of surgery or chemotherapy.

**Conclusions:**

Young people make up a small minority of patients with colorectal cancer. Young patients present with more locally advanced colorectal cancer. Despite this, on a population basis, their prognosis may be more favorable than their older counterparts when controlling for disease, patient, and treatment factors.

## Background

Colorectal cancer (CRC) is the most common malignancy of the gastrointestinal tract, the third most common cancer overall in Canada [1], and the second leading cause of cancer-related death. Colorectal cancer is typically a disease of the elderly, with over 90% of cases in patients >55 years of age [2]. The vast majority of cases in young patients (<40 years of age) are sporadic, as only 16% of young patients have been reported to have a predisposing factor and 23% to have a positive family history [3].

Whether young patients with CRC have a different biological behavior and carry a poorer prognosis remains controversial. Although younger patients are more likely to have mucinous or poorly differentiated tumors, including signet ring tumors [3, 4], studies have shown mixed results in terms of prognosis. Some studies suggested that younger age was a poor prognostic factor [5–7]. Other studies have suggested that this is not the case and young patients do at least as well, and possibly better, than their older counterparts [8–12]. The contradictory results of the prior studies may be due to inclusion of study subjects from single referral centers and/or lack of adjustment for potential confounding factors.

The objective of the current study was to determine the effect of age at diagnosis on the survival following treatment of CRC in the Province of Manitoba, Canada, while controlling for disease factors and treatment factors. This was truly a population-based study, avoiding some of the biases of single center studies.

## Methods

This study was approved by the University of Manitoba Health Research Ethics Board. It was a population-based historical cohort analysis of all adult patients (age ≥18 years) diagnosed with adenocarcinoma of the colon or rectum between 1 January 2004 and 31 December 2006 in the Province of Manitoba. Patients were identified using the Manitoba Cancer Registry (MCR), which contains information regarding all Manitobans diagnosed with a malignancy as mandated by Manitoba law [13]. The MCR was used to identify patients based on International Classification of Diseases-10 coding. Patients who were diagnosed with CRC at the same date as death, through autopsy or radiographic findings, were excluded (n = 3). From this registry, detailed demographic data including the age, gender, and postal code of the subjects’ home addresses was obtained. Socioeconomic status (income quintile) was assigned based on the mean household income in the neighborhood of residence, as determined from the 2006 Canadian Census data. The MCR also provided detailed tumor-specific information, including the histologic diagnosis and the TNM status at the time of diagnosis, and subsequent treatment information for each patient. This included dates of treatment, the specific surgical procedures performed, and receipt of chemotherapy or radiation therapy.

Information retrieved through this database was linked to other administrative databases maintained by Manitoba Health, the agency responsible for providing health insurance to virtually all citizens of the Province. The linkages were made using each patient’s Personal Health Identification Number, which was scrambled to maintain patient confidentiality. The Medical Claims (Physician Billing) Database contains patient-specific information about contacts with the healthcare system. This provided information about consultations and services provided to patients both in and out of hospital. These records identified the patient, the healthcare provider, and the location, type, and date of services rendered. In addition, the Hospital Separations Abstracts provided admission dates, discharge dates, and information on diagnoses and procedures, including complications of treatment. From these data, patient comorbidity was calculated according to a modification of the Charlson score [14–16] to allow this to be controlled in the analyses. The Manitoba Health Registry contains information on every Manitoban covered by the Manitoba healthcare insurance plan and provides up-to-date vital statistics information for each patient.

Young age was defined as being less than 45 years of age, and elderly patients were defined as being 80 years of age and above. Five-year overall survival was determined from the date of diagnosis. The survival analysis included a follow-up period lasting 5 years after the date of diagnosis or until 30 June 2011 (for those patients without 5 years follow-up). Because geography may influence treatment when patients must travel large distances to receive highly specialized care [17], the distance needed to travel to CancerCare Manitoba (the single tertiary care center in the province with most of the medical oncologists and all of the radiation oncologists in the province in the study time period) was included in the analysis. Patients were put into groups based on the distance required for travel (<21 km, 21 to 100 km, 101 to 500 km, and 501+ km). Geographical distance to CancerCare Manitoba was determined using SAS statistical software (SAS Institute Inc., Cary, NC, USA) and the Statistics Canada Postal Code Conversion File (PCCF+ Version 5G; Statisitcs Canada, Health Analysis Division. Ottawa, Ontario, K1A 0T6).

Standard descriptive analysis and treatment frequency are reported. Kaplan-Meier 5-year survival and a Cox proportional hazards model were performed. Two-sided *P* values were reported with significance set at *P* = 0.05. SAS statistical software versions 9.1 and 9.2 (SAS Institute Inc.) were utilized for statistical analyses.

## Results

Between 1 January 2004 and 31 December 2006 a cohort of 2,086 patients were diagnosed with colorectal adenocarcinomas in the Province of Manitoba. The median age of the entire cohort was 72 years, with range of 20 to 103 years. There was a slight male predominance of 53.8%. Seventy patients (3.36%) were considered young (45 years of age or younger). The patient demographics are listed in Table 1. Young patients were more likely to have T4 tumors and node-positive disease. Older patients had more advanced comorbidities.

**Table 1:** **Patient demographics**

	Age group			
	Young (<45 years)	Intermediate (45–79 years)	Elderly (80+ years)	
	n = 70	n = 1459	n = 557	***P***value
Diagnosis year (n (%))				0.1349
2004	28 (40.00)	502 (34.41)	176 (31.60)	
2005	26 (37.14)	451 (30.91)	194 (34.83)	
2006	16 (22.86)	506 (34.68)	187 (33.57)	
Age (mean ± SD (range))	38 ± 4.82 (20-44)	66 ± 9.21 (45-79)	85 ± 4.08 (80-103)	
Age (median (interquartile range)	39 (36-41)	67 (59-74)	84 (82-87)	<0.0001
Gender (n (%))				<0.0001
Female	35 (50.00)	607 (41.60)	321 (57.63)	
Male	35 (50.00)	852 (58.40)	236 (42.37)	
Site (n (%))				<0.0001
Colon	51 (72.86)	912 (62.51)	413 (74.15)	
Rectosigmoid	2 (2.86)	144 (9.87)	56 (10.05)	
Rectum	17 (24.29)	403 (27.62)	88 (15.80)	
AJCC stage (n (%))				<0.0001
I	9 (12.86)	296 (20.29)	98 (17.59)	
II	25 (35.71)	369 (25.29)	181 (32.50)	
III	22 (31.43)	449 (30.77)	139 (24.96)	
IV	13 (18.57)	319 (21.86)	107 (19.21)	
Unknown/NA	1 (1.43)	26 (1.78)	32 (5.75)	
T stage (n (%))				0.0267
NA		1 (0.07)		
T0		1 (0.07)	2 (0.36)	
T1	9 (12.86)	182 (12.47)	72 (12.93)	
T2	6 (8.57)	191 (13.09)	55 (9.87)	
T3	33 (47.14)	771 (52.84)	284 (50.99)	
T4	20 (28.57)	234 (16.04)	84 (15.08)	
TX	2 (2.86)	79 (5.41)	58 (10.41)	
Tis			2 (0.36)	
Nodes (n (%))				0.0058
N0	37 (52.86)	806 (55.24)	345 (61.94)	
N1	19 (27.14)	397 (27.21)	123 (22.08)	
N2	13 (18.57)	232 (15.90)	60 (10.77)	
NA		1 (0.07)		
NX	1 (1.43)	23 (1.58)	29 (5.21)	
Metastasis (n (%))				0.1626
M0	56 (80.00)	1124 (77.04)	436 (78.28)	
M1	13 (18.57)	319 (21.86)	107 (19.21)	
MX	1 (1.43)	15 (1.03)	14 (2.51)	
NA		1 (0.07)		
Grade/differential (n (%))				0.0038
1 - well differentiated	2 (2.86)	87 (5.96)	35 (6.28)	
2 - moderately differentiated	45 (64.29)	1003 (68.75)	329 (59.07)	
3 - poorly differentiated	8 (11.43)	180 (12.34)	75 (13.46)	
4 - undifferentiated		4 (0.27)	4 (0.72)	
9 - not determined/not available	15 (21.43)	185 (12.68)	114 (20.47)	
Residence at diagnosis (n (%))				0.0275
Rural	23 (32.86)	572 (39.20)	184 (33.03)	
Urban	47 (67.14)	887 (60.80)	373 (66.97)	
Distance from CancerCare (n (%))				0.0300
<21 km	44 (62.86)	835 (57.23)	341 (61.22)	
21-100 km	11 (15.71)	254 (17.41)	71 (12.75)	
101-500 km	14 (20.00)	324 (22.21)	138 (24.78)	
501+ km	1 (1.43)	46 (3.15)	7 (1.26)	
Charlson score (CCI) (n (%))				<0.0001
CCI count =0	39 (55.71)	604 (41.40)	194 (34.83)	
CCI count =1	29 (41.43)	558 (38.25)	178 (31.96)	
CCI count >1	2 (2.86)	297 (20.36)	185 (33.21)	
Socioeconomic status (n (%))				<0.0001
1	27 (38.57)	275 (18.85)	160 (28.73)	
2	11 (15.71)	318 (21.80)	125 (22.44)	
3	9 (12.86)	323 (22.14)	105 (18.85)	
4	10 (14.29)	289 (19.81)	75 (13.46)	
5	13 (18.57)	246 (16.86)	76 (13.64)	
NA		8 (0.55)	16 (2.87)	

AJCC (American Joint Committee on Cancer); CCI (Charlson Comorbidity Index); MX (M status cannot be assessed); NA (Not Available); Tis (Carcinoma in situ); Tx (T status cannot be assessed).

The treatments received by patients are listed according to age in Table 2 and according to stage in Table 3. Patients younger than 45 years were significantly more likely to receive chemotherapy, whereas elderly patients (≥80 years) were significantly less likely to receive surgery, chemotherapy, or radiation.

**Table 2:** **Treatments received according to age group**

	Age group			
	<45 years	45-79 years	80+ years	
	n = 70	n = 1459	n = 557	***P***value
Surgery type (n (%))				0.0001
Local resection	3 (4.29)	40 (2.74)	15 (2.69)	
Major surgery	55 (78.57)	1166 (79.92)	407 (73.07)	
None	10 (14.29)	196 (13.43)	124 (22.26)	
Polypectomy	2 (2.86)	57 (3.91)	11 (1.97)	
Chemotherapy type (n (%))				<0.0001
Adjuvant	34 (48.57)	509 (34.89)	35 (6.28)	
Local procedure	3 (4.29)	19 (1.30)	1 (0.18)	
Neoadjuvant	2 (2.86)	49 (3.36)	2 (0.36)	
None	24 (34.29)	804 (55.11)	512 (91.92)	
Palliative	7 (10.00)	78 (5.35)	7 (1.26)	
Radiation therapy type (n (%))				<0.0001
Adjuvant	6 (8.57)	159 (10.90)	16 (2.87)	
Local procedure	1 (1.43)	14 (0.96)	1 (0.18)	
Neoadjuvant	1 (1.43)	57 (3.91)	3 (0.54)	
None	61 (87.14)	1196 (81.97)	529 (94.97)	
Palliative	1 (1.43)	33 (2.26)	8 (1.44)

**Table 3:** **Treatments received according to stage**

	Stage					
	I	II	III	IV	Unknown/NA	
	n = 403	n = 575	n = 610	n = 439	n = 59	***P***value
Age group (n (%))						<0.0001
45-79	296 (73.45)	369 (64.17)	449 (73.61)	319 (72.67)	26 (44.07)	
80+	98 (24.32)	181 (31.48)	139 (22.79)	107 (24.37)	32 (54.24)	
<45	9 (2.23)	25 (4.35)	22 (3.61)	13 (2.96)	1 (1.69)	
Surgery type (n (%))						<0.0001
Local resection	29 (7.20)	12 (2.09)	10 (1.64)	6 (1.37)	1 (1.69)	
Major surgery	290 (71.96)	527 (91.65)	575 (94.26)	225 (51.25)	11 (18.64)	
None	30 (7.44)	33 (5.74)	19 (3.11)	204 (46.47)	44 (74.58)	
Polypectomy	54 (13.40)	3 (0.52)	6 (0.98)	4 (0.91)	3 (5.08)	
Chemo type (n (%))						<0.0001
Adjuvant	7 (1.74)	127 (22.09)	307 (50.33)	135 (30.75)	2 (3.39)	
Local procedure	1 (0.25)	6 (1.04)	11 (1.80)	5 (1.14)		
Neoadjuvant	2 (0.50)	17 (2.96)	26 (4.26)	8 (1.82)		
None	393 (97.52)	416 (72.35)	255 (41.80)	219 (49.89)	57 (96.61)	
Palliative		9 (1.57)	11 (1.80)	72 (16.40)		
Radiation therapy type (n (%))						<0.0001
Adjuvant	5 (1.24)	49 (8.52)	111 (18.20)	16 (3.64)		
Local procedure	3 (0.74)	4 (0.70)	8 (1.31)	1 (0.23)		
Neoadjuvant	2 (0.50)	19 (3.30)	35 (5.74)	5 (1.14)		
None	392 (97.27)	493 (85.74)	451 (73.93)	394 (89.75)	56 (94.92)	
Palliative	1 (0.25)	10 (1.74)	5 (0.82)	23 (5.24)	3 (5.08)	

**Figure 1:**
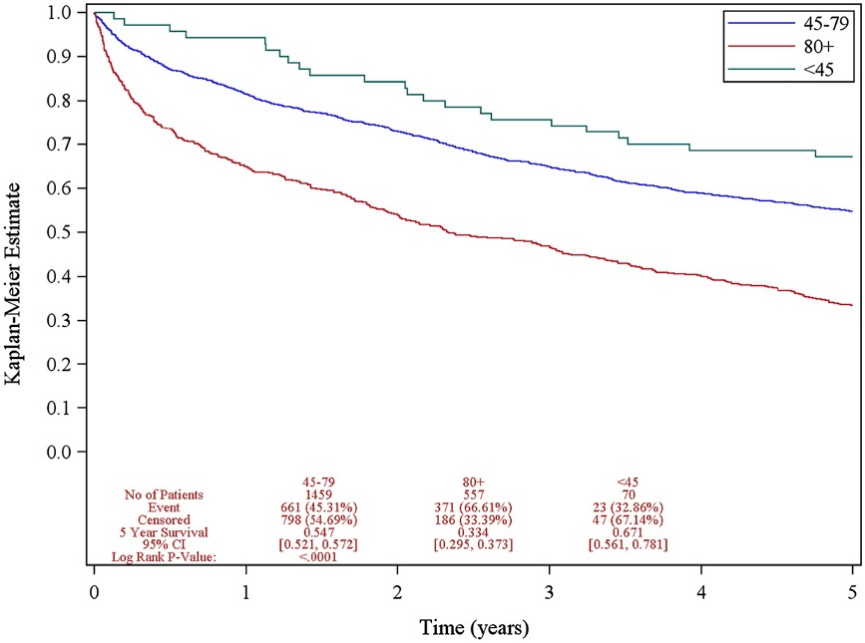
**Overall survival according to age.**

Figure 1 demonstrates the overall 5-year survival for the entire cohort according to age. The overall 5-year survival for patients under 45 years, patients aged 45 to 80 years, and patients 80 years and older were 67.1%, 54.7%, and 33.4%, respectively. Advanced age was associated with decreased overall survival. In this analysis, younger patients were found to have a superior overall survival relative to their older counterparts. The predictive factors for overall survival on univariate analysis are shown in Table 4 and the multivariate analysis is shown in Table 5. Young age remained a significant independent predictor of better survival while controlling for tumor factors, patient factors, and treatment factors. Poorer survival was associated with male gender, increasing stage, higher grade, increased comorbidity, lower socioeconomic status, and lack of receipt of surgery or chemotherapy.

**Table 4:** **Predictors of 5-year survival on univariate analysis**

	Event (n (%))	Hazard ratio	95% CI	***P***value
Diagnosis age group				<0.0001
45-79	661 (45.31)	1.54	1.02-2.34	
80+	371 (66.61)	2.87	1.89-4.38	
<45	23 (32.86)	1	Ref	
Gender				0.4521
Female	478 (49.64)	1	Ref	
Male	577 (51.38)	1.05	0.93-1.18	
Site				0.0069
Colon	719 (52.25)	1	Ref	
Rectosigmoid	103 (50.99)	0.93	0.75-1.14	
Rectum	233 (45.87)	0.79	0.68-0.91	
Collaborative stage				<0.0001
I	116 (28.78)	1	Ref	
II	194 (33.74)	1.21	0.96-1.53	
III	284 (46.56)	1.87	1.50-2.32	
IV	413 (94.08)	8.46	6.85-10.44	
Unknown/NA	48 (81.36)	6.7	4.78-9.39	
Income quintile				<0.0001
1	276 (59.74)	1	Ref	
2	244 (53.74)	0.88	0.74-1.05	
3	209 (47.83)	0.73	0.61-0.88	
4	165 (44.12)	0.66	0.54-0.80	
5	139 (41.49)	0.61	0.50-0.75	
NF	22 (91.67)	2.75	1.78-4.24	
Grade/differentiated				<0.0001
1 - well differentiated	43 (34.68)	1	Ref	
2 - moderately differentiated	624 (45.32)	1.39	1.02-1.89	
3 - poorly differentiated	152 (57.79)	2.18	1.56-3.06	
4 - undifferentiated	5 (62.50)	3	1.19-7.57	
9 - not determined/not available	231 (73.57)	3.65	2.64-5.06	
Comorbidity				<0.0001
CCI count <1	239 (28.55)	1	Ref	
CCI count =1	454 (59.35)	2.74	2.34-3.20	
CCI count >1	362 (74.79)	4.28	3.63-5.04	
Residence				0.4599
Rural	404 (51.86)	1	Ref	
Urban	651 (49.81)	0.95	0.84-1.08	
Distance from CancerCare				0.348
<21 km	599 (49.10)	1	Ref	
21-100 km	170 (50.60)	1.02	0.86-1.20	
101-500 km	257 (53.99)	1.12	0.97-1.29	
501+ km	29 (53.70)	1.25	0.86-1.81	
Surgery type				<0.0001
Major surgery	707 (43.43)	0.17	0.15-0.19	
Local resection	15 (25.86)	0.09	0.05-0.15	
Polypectomy	27 (38.57)	0.14	0.10-0.21	
None	306 (92.73)	1	Ref	
Chemotherapy type				<0.0001
None	690 (51.49)	1	Ref	
Neoadjuvant	19 (35.85)	0.54	0.34-0.85	
Adjuvant	254 (43.94)	0.71	0.62-0.82	
Palliative	83 (90.22)	2.53	2.01-3.18	
Adjuvant with local procedure	9 (39.13)	0.61	0.32-1.18	
Radiation therapy type				<0.0001
None	929 (52.02)	1	Ref	
Neoadjuvant	18 (29.51)	0.45	0.28-0.71	
Adjuvant	65 (35.91)	0.56	0.43-0.71	
Palliative	39 (92.86)	2.67	1.94-3.69	
Adjuvant with local procedure	4 (25.00)	0.38	0.14-1.00	

CCI (Charlson Comorbidity Index); NA (Not Available); NF (Not Formatted).

**Table 5:** **Multivariate Cox proportional hazards model of 5-year overall survival**

	Event (n(%))	Adjusted hazard ratio	95% CI	***P***value
Diagnosis age group				<0.0001
45-79	661 (45.31)	1.29	0.85-1.97	
80+	371 (66.61)	1.95	1.27-3.01	
<45	23 (32.86)	1	Ref	
Gender				0.0045
Female	478 (49.64)	1	Ref	
Male	577 (51.38)	1.2	1.06-1.36	
Collaborative stage				<0.0001
I	116 (28.78)	1	Ref	
II	194 (33.74)	1.22	0.96-1.56	
III	284 (46.56)	1.72	1.34-2.20	
IV	413 (94.08)	6.12	4.73-7.91	
Unknown/NA	48 (81.36)	2.48	1.71-3.59	
Income quintile				0.0003
1	276 (59.74)	1	Ref	
2	244 (53.74)	1.09	0.91-1.30	
3	209 (47.83)	0.95	0.79-1.14	
4	165 (44.12)	0.91	0.75-1.11	
5	139 (41.49)	0.74	0.60-0.91	
NF	22 (91.67)	1.89	1.21-2.95	
Grade/differentiated				0.0004
1 - well differentiated	43 (34.68)	1	Ref	
2 - moderately differentiated	624 (45.32)	1.15	0.84-1.58	
3 - poorly differentiated	152 (57.79)	1.59	1.13-2.25	
4 - undifferentiated	5 (62.50)	1.93	0.76-4.92	
9 - not determined/not available	231 (73.57)	1.52	1.08-2.14	
Comorbidity				<0.0001
CCI count <1	239 (28.55)	1	Ref	
CCI count =1	454 (59.35)	2.06	1.72-2.47	
CCI count >1	362 (74.79)	2.9	2.41-3.49	
Surgery type				<0.0001
Major surgery	707 (43.43)	0.32	0.26-0.40	
Local resection	15 (25.86)	0.18	0.09-0.35	
Polypectomy	27 (38.57)	0.51	0.33-0.80	
None	306 (92.73)	1	Ref	
Chemotherapy type				<0.0001
None	690 (51.49)	1	Ref	
Neoadjuvant	19 (35.85)	0.76	0.48-1.22	
Adjuvant	254 (43.94)	0.6	0.50-0.72	
Palliative	83 (90.22)	0.4	0.30-0.52	
Adjuvant with local procedure	9 (39.13)	1.08	0.47-2.51	

CCI (Charlson Comorbidity Index); NA (Not Available); NF (Not Formatted).

## Discussion

While the incidence of CRC cases in older adults has remained stable, the incidence in young adults (aged 20 to 40 years) has been steadily increasing [18]. From 1973 to 1999, the Surveillance, Epidemiology, and End Results registry indicated that colon and rectal cancers increased by 17% and 75%, respectively, in persons aged 20 to 40 years, whereas the rates in those 50 years and older remained stable or declined. CRC is one of the top ten most common cancers amongst those aged 20 to 49 years, and for patients in their third and fourth decades CRC is one of the top four most common cancers [19].

Despite having many poor prognostic factors, young patients in this population-based study had better 5-year overall survival both in the univariate (unadjusted) and multivariate analysis. This study provides contemporary data for discussion for newly diagnosed young patients with CRC and their healthcare providers.

In this study, younger patients were found to have more locally advanced tumors at the time of diagnosis. They had a greater proportion of T4 tumors as well as node-positive tumors. Others have found similar findings with younger patients having higher stage disease [4, 20, 21] and less favorable histologic subtypes than older patients [21–23]. It is not clear why younger patients tend to present with more advanced disease. Perhaps, because of their young age, physicians are less likely to suspect malignant disease than they are older patients, thus leading to a delay in appropriate investigations and diagnosis. Another potential explanation is that younger patients would generally not be included in CRC screening initiatives and would be less likely to have early cancers diagnosed through these screening initiatives. It was not possible to determine whether cancers were detected through screening from the available databases.

Despite these poor prognostic factors, the outlook in this group of patients is actually more favorable. When controlling for tumor, patient, and treatment factors, young patients had a superior 5-year overall survival. This is in contrast to some older studies that suggested young patients had a worse prognosis [5, 7, 8]. Other more recent studies have suggested that the survival is no different when adjusting for confounding variables [9–12].

In this study, young age was defined as 45 years and under. CancerCare Ontario and the Candian Cancer Society have previously used this definition [24]. The proportion of young patients was small at 3.36%, in keeping with other studies [4, 15, 25]. Expanding the definition of young age to include patients who were 50 years and under would have captured as many as 13% of patients [25]. This would improve the statistical power of the analysis, but potentially lower the clinical relevance of the possible influence of a different biological behavior among colon cancers in younger patients; this would have been diluted by including many slightly older patients carrying cancers with a more typical biology.

In this study, tumor stage and treatment variables were controlled for; despite this, however, it is likely that younger patients were treated more aggressively. This may partly explain their improved prognosis. This analysis was able to adjust for the receipt of chemotherapy (or not), but it was unable to control for differences in chemotherapuetic agents and duration of treatment/discontinuation of treatment. With recent advances in chemotherapy for CRC, it is possible that younger patients were treated with more aggressive, but potentially more toxic, regimens compared to older patients. It is also possible that older patients were more likely to discontinue chemotherapy early due to side effects or other reasons. The databases used in this study do not collect information regarding performance status, which would have been another important variable in selecting older patients for more aggressive systemic treatment.

Another possible explanation for the improved prognosis is that this analysis used overall survival as the primary outcome measure, rather than cancer-specific survival. It was felt that this a more robust outcome measure that would be less sensitive to biases in determining cause of death. Since younger patients are expected to have fewer comorbidities and fewer competing causes of death, this could also contribute to their more favorable 5-year overall survival. Although the multivariate analysis did adjust for the burden of comorbid disease, this adjustment may not have been complete.

A limitation of the study is that younger patients may have had a higher proportion of hereditary CRC syndromes than older patients. This could not be evaluated with the available data. Furthermore, important pathologic features such as microsatellite instability or results or immunohistochemistry for DNA mismatch repair abnormalities are not captured in the MCR. Although studies suggest that only a small minority of young patients with CRC have genetic or other predisposing factors [3, 21], it is likely that a higher proportion of patients with factors such as hereditary nonpolyposis CRC existed in the younger cohort. Since hereditary nonpolyposis CRC tumors are associated with a better prognosis [26–28], this could partially be responsible for this study’s findings.

The biggest strength of this paper is that it is a population-based analysis, and examines all patients whether they had surgical resection or not. Thus, it avoids some of the biases associated with series of selected patients from single institution referral centers.

## Conclusions

Young people make up a small minority of patients with CRC. Young patients present with more locally advanced disease at diagnosis. Their prognosis is more favorable than their older counterparts when controlling for disease, patient, and treatment factors.

## Abbreviations

CRC: colorectal cancer
MCR: Manitoba Cancer Registry.
